# Effect of Salinity and Polycarboxylate Superplasticizer on Fresh Property of Seawater-Blended Cement

**DOI:** 10.3390/polym15030541

**Published:** 2023-01-20

**Authors:** Jun Ren, Hao Li, Ji Zhang, Shuo Yan, Haiyan Zhu, Shengye Xu, Shi Shi, Jianghong Mao

**Affiliations:** 1 Urban Construction and Digital City Teaching Experiment Center, School of Architecture and Planning, Yunnan University, Kunming 650550, China; 2 Guangdong Power Grid Energy Development Co., Ltd., Guangzhou 510800, China; 3 Department of Civil, Environmental and Geomatic Engineering, University College London, London WC1E 6BT, UK; 4 School of Architecture and Environment, Sichuan University, Chengdu 610207, China

**Keywords:** polycarboxylate superplasticizer, seawater-mixed cement, salinity, rheology, molecular structure

## Abstract

The salinity of seawater can affect the properties of mixtures of polycarboxylate superplasticizer (PCE) and seawater. The purpose of this research is to study the effect of different salinities of water on the property of seawater-mixed cement slurry. Two PCE types with different side chain lengths and acid–ether ratios were used. Their physicochemical properties were explained by nuclear magnetic resonance (NMR), size exclusion chromatography (SEC), Fourier transform infrared spectroscopy (FTIR), turbidimetry, and dynamic light scattering (DLS) analysis. The performance of the PCEs was measured using slump, rheological energy, and solidification time. Finally, by measuring the adsorption volume, adsorption layer thickness, and water film thickness (WFT), the mechanisms involved in performance modification were studied. The results show that the workability and rheological performances of seawater-mixed cement paste are decreased by increasing salinity. With the increase in salinity, the minislump of the seawater-blended cement pastes with two PCEs decreased from 285 mm to 120 mm and from 280 mm to 78 mm, respectively, and the thixotropic areas were increased from less than 2000 Pa/s to above 10,000 Pa/s. Moreover, the adsorption amount of the two PCEs in the cement mixed with high-salinity seawater decreased by 55.99% and 71.56%, respectively, and the thickness of the adsorption layer and water film was decreased with increasing salinity. Compared with the two PCEs, PCE with long side chains and a high acid–ether ratio provided better salt resistance.

## 1. Introduction

Among commonly used building materials, concrete has seen the fastest growth in consumption over the past 50 years [1]. The huge associated energy consumption has focused attention on the sustainable development aspects of concrete [2,3,4], and many studies have been carried out on the sustainable utilization of the raw materials that can be used in the manufacture of concrete. As a matter of common knowledge, readily available materials such as clay and industrial waste have been used to replace cement [5], and construction waste has been used to make recycled aggregate [6], which greatly reduces resource consumption. However, in addition to cement and aggregate, water as a component of concrete has not been considered in terms of sustainability. It has been estimated that global annual water consumption for concrete production accounts for 18% of global annual industrial water consumption [7]. Seawater (SW) is inexhaustible; its use results in zero carbon emissions compared to the use of tap water (TW), and the utilization of SW in manufacturing concrete is an effective way to reduce the unnecessary use of freshwater resources [8].

In recent years, many scholars have carried out research on seawater-mixed concrete. Seawater-mixed concrete has better strength than ordinary concrete [9], but SW is rich in chloride (Cl^−^), sodium (Na^+^), sulfate (SO_4_^2−^), magnesium (Mg^2+^), and other substances, which can lead to ion penetration and reinforcement corrosion [10,11,12]. Although seawater-mixed concrete can be used for unreinforced concrete to avoid ion corrosion [13], the higher viscosity of seawater-mixed concrete limits its wide application. Compared with ordinary concrete, the workability of seawater-mixed concrete is reduced by about 30% [14], and the setting time is also significantly reduced [15]. This is because the rich Cl^−^ in SW can precipitate during the hydration of C_3_S and C_3_A [16]. Other ions present in SW, such as SO_4_^2−^, Mg^2+^, and calcium (Ca^2+^), also affect the hydration of cement [17,18]. These ions accelerate the hydration process, but also reduce the workability of cement. Wang et al. [17] studied the rheological behavior of seawater-mixed mortar and deionized-water-mixed mortar. The shear stress and viscosity of seawater-mixed mortar are significantly higher. The reason for this is the stronger flocculation between the larger solid content and the suspended particles produced by the accelerated hydration of SW. In addition, according to the influence of ions on seawater-mixed concrete, it is essential to compensate and ensure its adequate fluidity by adding a high-efficiency water-reducing agent [19,20].

Polycarboxylate (PCE) is a comb polymer with a strong molecular structure design, and it is considered to be the best dispersing agent for cement-based materials [21]. Based on the structure of PCE comb copolymer with both carboxyl and polyethylene glycol (PEO) side chains, PCE copolymer produces electrostatic repulsion and steric hindrance, so it has excellent performance including long-term retention, higher water reduction rate, and stronger environmental protection [22,23]. At the same time, because of the strong designability of its molecular structure, in addition to its water-reducing type, polycarboxylate superplasticizers (PCEs) with different functions such as slump retention, early strength, and mud resistance have also been derived to meet different construction environments and sand quality requirements [24]. However, at present, no PCE suitable for high-salinity environments has been put to use due to the different ion strength in seawater. Since the salinity of seawater is different in different areas, it is of great theoretical and practical value to study the effect of different SW salinity on PCE in seawater-mixed concrete.

The adsorption of PCE on cement particles needs to interact with ions on the surface of cement particles. Therefore, the ions in suspensions are important factors affecting adsorption [25]. PCE in SW is affected by Ca^2+^, Mg^2+^, and SO_4_^2−^ ions, and its dispersion is inhibited [26,27]. It has been reported that, with the increase in SO_4_^2−^ concentration, the adsorption rate of PCE and the fluidity of cement paste decrease because SO_4_^2−^ and PCE are competitively adsorbed [28,29]. PCEs with higher carboxylic density may significantly enhance sorption. On the other hand, Mg^2+^ ions promote the adsorption of polycarboxylic by forming complexes with carboxylate groups, and this effect is negatively correlated with the concentration of polycarboxylic [30]. Hence, the use of low-charge-density PCEs may achieve better adsorption effect. Based on the above, the adsorption of PCE in SW is closely related to the concentration of SW ions and the type of PCE used [31,32]. Although there has been much research on the influence from different ions in seawater on concrete properties, most of the research is based on the effect of a certain type or levels of ions. In fact, the combination of different ions could make the situation more complex, therefore requiring the investigation on the effect of the salinity (or the concentration of ions) in the performance of PCEs.

At present, the characterization of PCE in SW of different salinities and the mechanisms controlling how SW and PCE interact remain to be studied. In order to explore the effects of PCE with different side chain lengths and acid–ether ratios on SW cement paste with different salinity, the following work was carried out in this study: First, synthesized PCE is characterized using nuclear magnetic resonance (NMR), size exclusion chromatography (SEC), Fourier transform infrared spectroscopy (FTIR), nephelometry, and dynamic light scattering (DLS) analysis. The associated mechanisms are then explored by analyzing changes in the adsorption capacity, adsorption layer thickness, and water film thickness (WFT).

## 2. Materials and Methods

### 2.1. Materials

Standard cement in accordance with the Chinese National Standard GB8076-2008 with a specific surface area of 355 m^2^/kg was provided from Aosaier Co., Ltd. (Fushun, China). The chemical composition of the cement was determined by XRF and the quantitative XRD Rietveld method, the results of which are shown in Table 1.

The reagents used to synthesize the PCEs used in the study, such as persulfate (KPS) and acrylic acid (AA), were supplied by Tianjin Fuchen Chemical Reagents Manufacture (Tianjin, China).

Two types of methyl allyl polyoxyethylene ethers (HPEGs), with molar mass of around 2400 g·mol^−1^ (EO52) and 3000 g·mol^−1^ (EO66), were used as the macromonomer, and these were manufactured by the Jilin Zhongxin Chemical Group Co., Ltd. (Jilin, China).

The tap water (TW) used in this experiment to make cementitious materials was ordinary mains-supplied water. Medium-salinity SW (MSW) was collected from Dapeng Bay in Shenzhen, China. The MSW was diluted with deionized (DI) water to simulate lower-salinity SW (LSW), while it was concentrated by heating to provide higher-salinity SW (HSW). Production of the SWs followed the methods of Wang et al. [33]. The ion concentration of water was determined by inductively coupled plasma emission spectrometer (ICP), the results of which are presented in Table 2. 

### 2.2. PCE Synthesis

**Figure 1:**
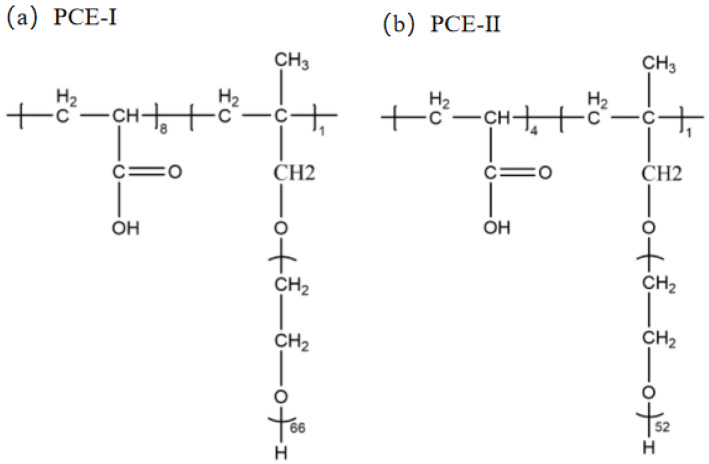
Molecular structure of the PCEs used in this study. (**a**) PCE-I; (**b**) PCE-II. See Table 3 for their monomer molar ratio and solid content.

The synthesis parameters of the two PCEs synthesized and determined are presented in Table 3, and their molecular structures are shown in Figure 1. Using the synthesis method of Ren et al. [34], KPS was used as the initiator of free radical polymerization. After synthesis, the polymer solution was neutralized with 30 wt% sodium hydroxide solution.

### 2.3. PCE Characterization

The molecular constitution of the PCEs was determined using ^1^H-Nuclear Magnetic Resonance spectroscopy (^1^HNMR, AVANCE II1, Bruker, Karlsruhe, Germany). For the ^1^HNMR characterization, the PCEs were first dried in a vacuum at 40 °C until they reached a constant weight. Then, the dried PCE samples were dissolved in deuterium oxide (D_2_O) at 25 °C and characterized using a 500 MHz NMR spectrometer.

In order to determine the molecular weight and mass distribution, gel permeation spectrometry (GPC) was used according to Sun’s method [35], with 0.1 M NaNO_3_ as the eluent and a flow rate of 1.0 mL/min.

The functional groups of the PCEs were then determined using a Fourier transform infrared spectrometer with an ATR mode (FTIR-ATR, PerkinElmer, Boston, MA, USA). FTIR spectra between 600 and 4000 cm^−1^ were obtained.

The turbidity of PCEs (with TW, LSW, MSW, and HSW) was measured using a digital turbidimeter (WGZ-20S, Shanghai Xinrui Instrument Co., Ltd., Shanghai, China), in which a standard silica solution was used for calibration. 

In order to analyze the configuration of PCEs in solutions of different salinities, the hydrodynamic radius (Rh) was tested by Dynamic Light Scattering. The characterization was conducted under a concentration of 1 mg/mL using an ALV machine with testing angle of 90° following the method of Shu et al. [36].

### 2.4. PCE Performance in Cementitious Materials

#### 2.4.1. Preparation

Following the Chinese standard GB8077-2012, the water cement ratio was 0.29, and the dosage of PCE was controlled to 0.15 wt% of the cement, which offered a sufficient workability for rheology tests. 

#### 2.4.2. Workability Test

The workability of cement paste was tested using the minimum slump of cement paste according to the national standard method GB/T 8077-2012. The propagation diameter of the minimum slump test was measured in two vertical directions, and the average diameter was calculated. The initial mini-slump was tested at 5 min, followed by the slump at 30 min and 60 min, and the change of slump over this time period was used to evaluate the working performance retention rate.

#### 2.4.3. Rheological Test

The variation of shear stress with shear rate of the cement paste was measured using a Lamy viscometer RM100 to observe its rheological properties. During measurement, the viscometer used a 5 s rest time. The shear rate increased from 2 to 200 s^−1^ and then decreased from 200 to 2 s^−1^, and the whole process lasted for 90 s. Values of 2, 10, 20, 40, 80, 120, 160, and 200 s^−1^ were selected to record the shear. According to the method of Chen [37], the thixotropic area of the cement paste was obtained by integrating. Since rheology leads to structural damage, different rheological models (Equations (1)–(3)) were fitted using flow curves following the methods of Ren et al. [38]. 

Bingham model:

(1) $$\tau =\tau_{0}+\mu \cdot \dot{\gamma }$$

Modified Bingham model:

(2) $$\tau =\tau_{0}+\mu \cdot \dot{\gamma }+c\cdot {\dot{\gamma }}^{2}$$

Herschel–Bulkley model:

(3) $$\tau =\tau_{0}+K\cdot {\dot{\gamma }}^{n}$$

where τ is the shear stress (Pa), $\tau_{0}$ is the yield stress (Pa), μ is the plastic viscosity (Pa·s), $\dot{\gamma }$ is the shear rate (s^−1^), c is the second order parameter (Pa·s^2^), K is the consistency factor (Pa·s^n^), and n is the exponent.

#### 2.4.4. Setting Time Test

According to the standard method GB/T1346-2011, the initial and final setting time of cement the paste were tested by Vicat apparatus.

#### 2.4.5. Compressive Strength Test

The cement pastes with size of 20 mm × 20 mm × 20 mm were used for a compressive strength test. The samples were tested after curing in a standard curing environmental chamber (20 °C and 95% RH) for 3, 7, 28, and 56 days. The tests were conducted three times, and the average values of the three specimens were recorded. The standard deviation of all values was controlled below 10%.

#### 2.4.6. Adsorption Amount Measurement

By analyzing the total organic carbon (TOC) using a Multi N/C 2100 instrument, the amount of PCEs adsorbed onto the particle surface of cement paste was determined. Following a commonly used procedure [38,39], a cement suspension consisting of 1 g of cement and 20 g solution (including TW, LSW, MSW, HSW, and PCEs) was first prepared and centrifuged at 700 rpm, stopped after 3 min, then separated by extraction and filtration. The adsorption capacity of PCE can be calculated by the change of PCE concentration [38].

#### 2.4.7. Adsorption Layer Thickness Measurement

X-ray photoelectron spectroscopy (XPS) was prepared by SEMEFER K-Alpha (SEMEFER GmbH in Massachusetts, USA) using dry solid powder. The thickness of the adsorption layer was then tested using XPS (Thermo Scientific K-Alpha, Thermo Fisher Ltd., Waltham, MA, USA). After a period of mixing, the suspension of cement paste with/without PCEs was dried in a vacuum. The sorption layer thickness of the PCEs may be calculated by Si2p analysis [40].

#### 2.4.8. Water Film Thickness Measurement

The WFT (Equation (4)) was estimated using the excess water content and specific surface area of the cement particles [41]. The excess water ratio content is calculated using the difference between the minimum pore ratio obtained by the wet accumulation measure and the volume ratio of water to solids:

(4) $$WFT\;=\;\frac{u_{w}^{\prime }}{A_{c}}$$

where *WFT* is the water film thickness, $u_{w}^{\prime }$ is the volume ratio of excess water to cement, and $A_{c}$ is the specific surface area of cement particles. The specific surface area of cement particles was calculated using the BET [42]. 

## 3. Results

### 3.1. PCE Characterization

#### 3.1.1. ^1^H NMR Analysis

**Figure 2:**
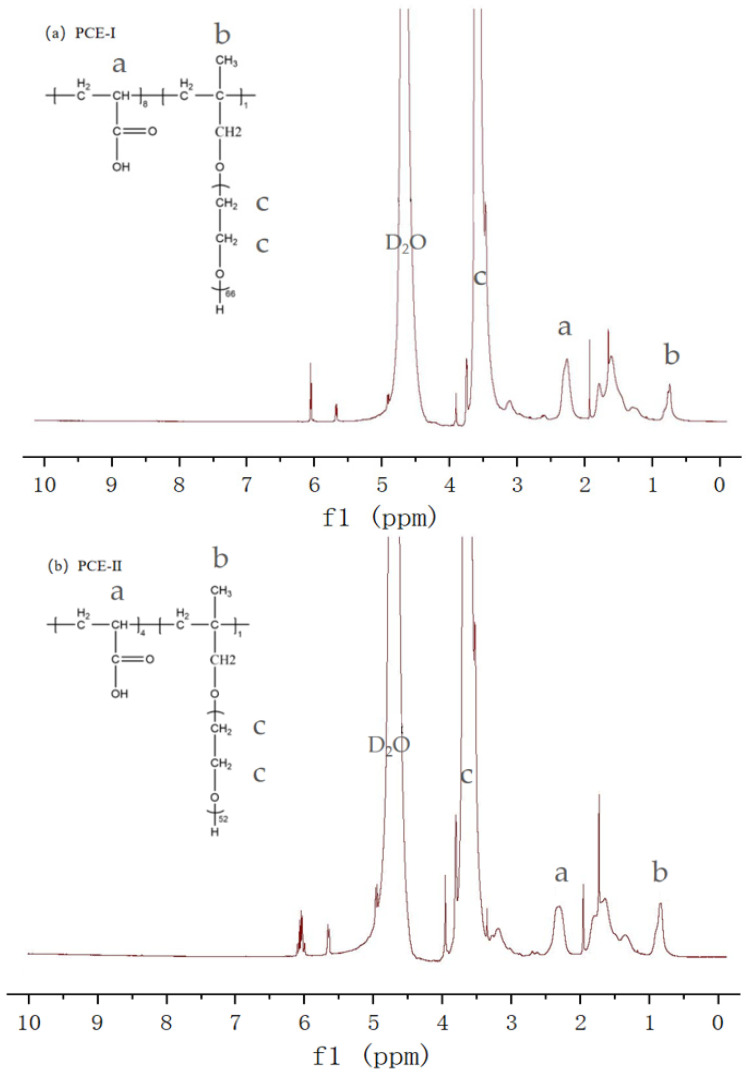
^1^HNMR of the PCEs used in this study. (**a**) PCE-I; (**b**) PCE-II.

The ^1^HNMR spectra of the two PCEs are presented in Figure 2. It can be seen from the figure that the molecular composition of the two PCEs is similar. The peak with a chemical shift from 0.6 to 0.9 ppm (Figure 2, Peak b) belongs to -CH_3_ from the HPEG in the PCE skeleton [43]. In addition, the strong peak at 3.0~4.0 ppm corresponds to the H atom (-CH_2_-CH_2_-O) of the PEO side chain (Figure 2, Peak c). Moreover, for the main chain, the -CH_2_, -CH- groups of acrylic acid peaked at about 2.3 ppm; this is because of the comb-like structure of the polymer.

#### 3.1.2. Molecular Weight and Weight Distribution

The molecular weights of PCE-I and PCE-II were obtained by GPC, and the results are summarized in Table 4. Molecular weight plays an important role in the performance of PCE. Too low a molecular weight leads to insufficient slump of concrete, and too high a molecular weight could decrease the dispersion of cement paste [44]. The Mn (30,125 and 24,416 Da) of the two PCEs was around 30,000 Da, which could provide a better performance in dispersing cement particles [45]. Moreover, the PDI (1.82 and 1.76) of the PCEs was below 2.00, which indicated a narrow molecular weight distribution [38]. 

#### 3.1.3. FTIR Analysis

**Figure 3:**
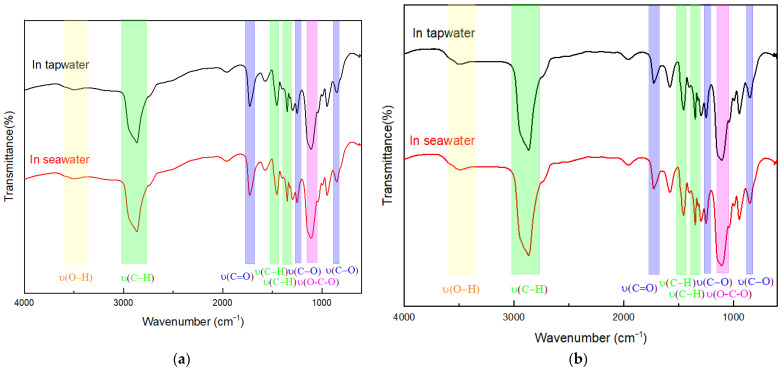
FTIR spectra of the two PCEs in TW or MSW. (**a**) PCE-I; (**b**) CPE-II.

The FTIR spectra of the PCEs are presented in Figure 3. The strong broad peak at around 3500 cm^−1^ is related to the vibration of O-H. It is known that PCE contains hydroxyl groups. Peaks at 2885, 1466, and 1341 cm^−1^ can be attributed to the methyl group from the backbone of the PCEs. In addition, the peaks at 1715, 1241, and 840 cm^−1^ can be ascribed to the stretching of the C-O bond, which confirms the existence of carboxylate groups. The asymmetric stretching vibration of C-O-C was observed at 1100 cm^−1^, further revealing the HPEG side chain of the PCE. Notably, no new peaks were observed in the FTIR spectra, suggesting that the salinity of the water did not cause structural changes in the PCEs. Therefore, the degradation of PCEs under the action of ions, leading to performance differences between PCEs in cement paste with different salinities, can be excluded.

#### 3.1.4. Turbidity

**Figure 4:**
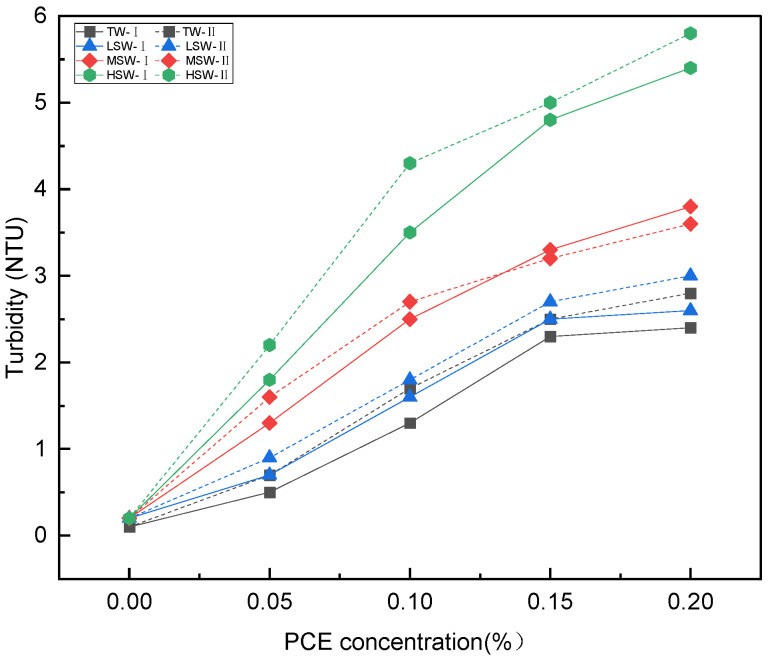
Turbidity of the PCEs in TW and SW.

In order to further investigate the solubility of the PCEs in different media, the turbidity of the PCE solution under different concentration was observed, and the results are plotted in Figure 4. Regardless of PCE type, the turbidity was significantly increased with increased PCE concentration. Generally, when the concentration was less than 0.15%, the turbidity increases rapidly with increasing PCE concentration, which the increment speed slowed after it reached 0.15%.

Turbidity increased with the increase of seawater salinity, which could be attributed to the instability of PCEs in seawater. However, no sudden point (cloudy point), at which the transparent polymer solution could be changed to turbid media, was observed, indicating that the solubility of the PCEs did not change [46]. For both of the PCEs, the lowest turbidity was observed in TW, which suggested that the best solubility was achieved in the PCEs. Correspondingly, the rich ions in HSW led to the poor stability of PCEs. Therefore, PCEs have the highest turbidity and lowest solubility in HSW.

#### 3.1.5. Hydrodynamic Radius Analysis

**Figure 5:**
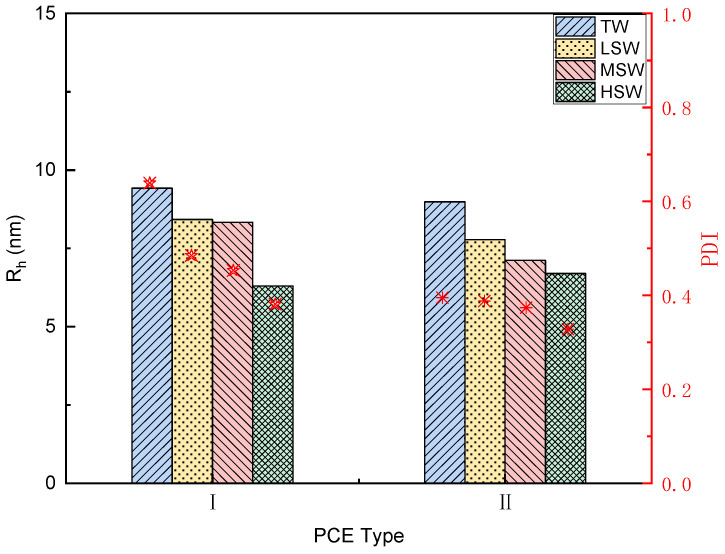
Hydrodynamic radius (Rh) and PDI values of the two PCE types in TW and SW.

The hydrodynamic radii (Rh), indicating the configuration of the PCEs in solution, and their PDI values are shown in Figure 5. The figure shows that replacement of TW with SW significantly reduced the Rh of the PCEs, which suggests that the steric space from the PCEs was blocked when it was dissolved in SW. The effects of salinity can be explained using Figure 5. The Rh of the PCEs was reduced with increasing salinity, indicating that the PCEs were transferred to a “rigid state”. The increased salinity introduced more ions (i.e., Na^+^, Cl^−^, SO_4_^2−^, or Mg^2+^) into the solution, and the hydrolysed carboxylate groups from the backbone of the PCE then reacted with those ions to form a complex, introducing a “salting-out” effect on the backbone [38,47]. On the other hand, the increased ion strength in the solution may have led to distortion and shrinkage of the PCE side chain, which further reduced the Rh. This assumption was confirmed by the PDI values under different salinities, as the strong ion strengths in high-salinity SW reduced the PDI values [38]. 

### 3.2. Workability

**Figure 6:**
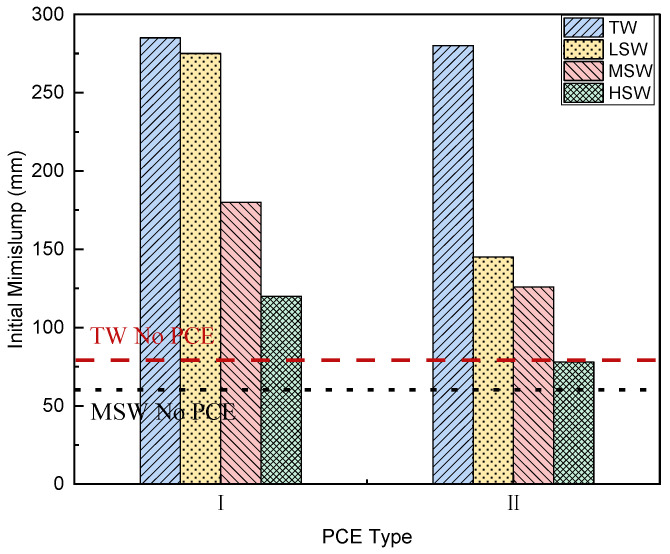
Effect of SW salinity on the initial minislump of cement paste with the two PCEs.

The workability of cement paste with different salinity added with PCEs was tested by a minislump test, and these values are presented in Figure 6. The minislump at 5, 30, and 60 min was measured, and the results are presented in Table 5 in order to evaluate workability retention.

As can be seen in Figure 6. that the addition of PCE increases the initial slump of cement paste in both TW and SW. Similar to other research, the effect of two types of PCE was similar in TW. The addition of PCE-I and PCE-II increased the minislump of cement paste from 80 mm to 285 mm and 280 mm, respectively. However, in MSW, the minislump of the cement paste increased from 60 mm to 180 mm after adding PCE-I, and this was significantly greater than the increase in PCE-II (126 mm).

Compared with the performance of PCE-II, PCE-I had better tolerance to ion action in SW, which may be due to the longer side chain and higher acid–ether ratio. As reported by Yang et al. [31], the PCE with long side chains and high acid–ether ratio showed less sensitivity with cement paste. Therefore, PCE with long side chains and high acid–ether ratio could be suitable for seawater concrete. Moreover, as presented in Figure 5, the lowest reduction in Rh was observed in PCE-I, indicating that the PCE-I itself did not significantly shrink in the presence of large ions in SW. Therefore, steric repulsion from PCE-I had no obvious effect.

It can be seen from Figure 6 that increased SW salinity further reduces the initial minislump of the cement paste. This is consistent with the results of Ren [38]. For example, with the replacement of TW with LSW, MSW, and HSW, the minislump of cement paste with PCE-I reduced from 285 mm to 275, 180, and 120 mm, respectively, while that incorporated with PCE-II reduced from 280 to 265, 126, and 78 mm, respectively. Thus, better performance was achieved by PCE-I. As the massive ions in SW, particularly the Cl^−^ and SO_4_^2−^ ions, introduced competitive adsorption of the PCEs [47], the PCE with high acid–ether ratio provided better resistance to this competitive adsorption. 

As shown in Table 5, the cement paste of the two PCEs in TW had good workability. However, when TW changed to SW, the minimum mass loss in the first 60 min increased significantly, and the loss value was more obvious when the ion concentration was higher. This may be due to the effects of the large number of ions in SW; the C3A dissolved faster, which accelerated the hydration of the SW-mixed cement. With more AFt production, the workability retention of the PCEs was hindered [30,31,32]. 

### 3.3. Rheological Behavior

#### 3.3.1. Thixotropic Behavior

**Figure 7:**
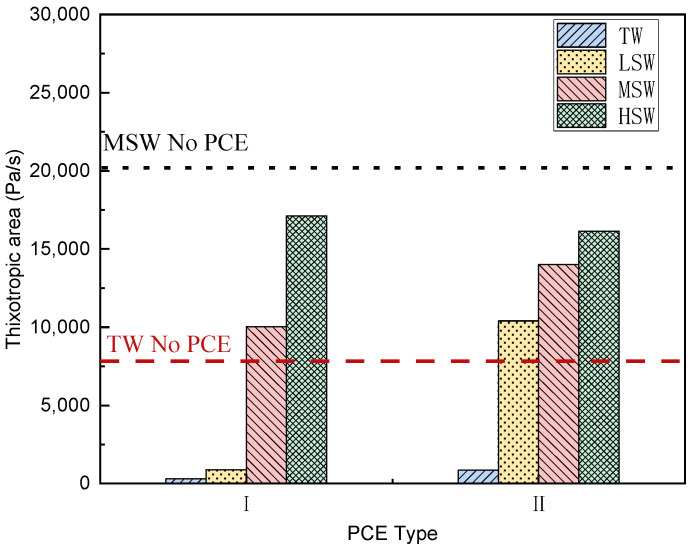
Effect of SW salinity on the thixotropic area of cement paste with the two PCEs.

The thixotropic area of the cement paste mixed with PCEs was calculated from the flow curves and is presented in Figure 7. It can be seen from Figure 7 that both PCE cement pastes have low thixotropy in TW. However, a significate increase in the thixotropic area was observed in SW-blended cement paste under all three salinities, suggesting that the SW-blended cement had a tendency of flotation. This could be because the massive ions in the SW, including Na^+^, Ca^2+^, Mg^2+^, Cl^−^, and SO_4_^2−^, led to a high-strength solution and made it difficult to de-flocculate the cement particles in the suspension. This is further confirmed by the increase in thixotropic area with increasing salinity. For example, the smallest thixotropic area of the cement paste with PCE-I of 880 Pa/s was observed in LSW, while it dramatically increased to over 10,000 Pa/s in MSW and HSW. 

#### 3.3.2. Rheological Parameters

**Figure 8:**
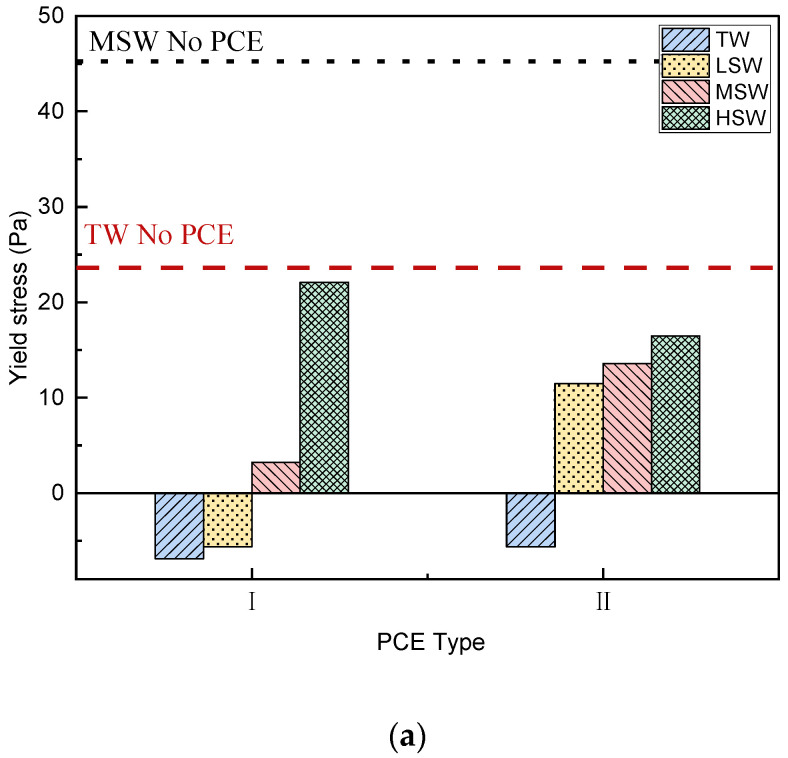

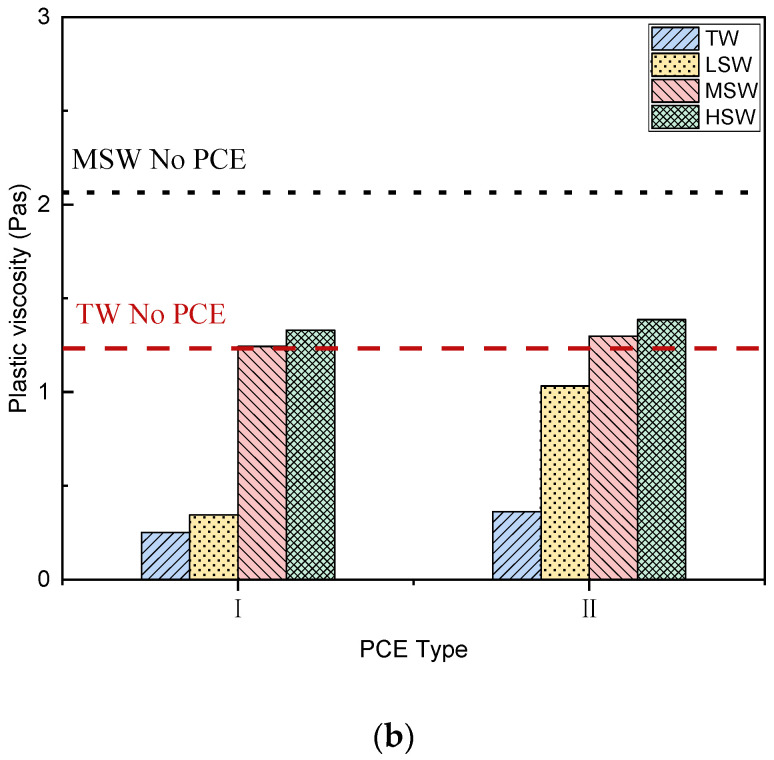
Effect of SW salinity fitted by the Bingham model on rheological behavior of cement paste with the two PCEs (Equation (1)). (**a**) Yield stress. (**b**) Plastic viscosity.

**Figure 9:**
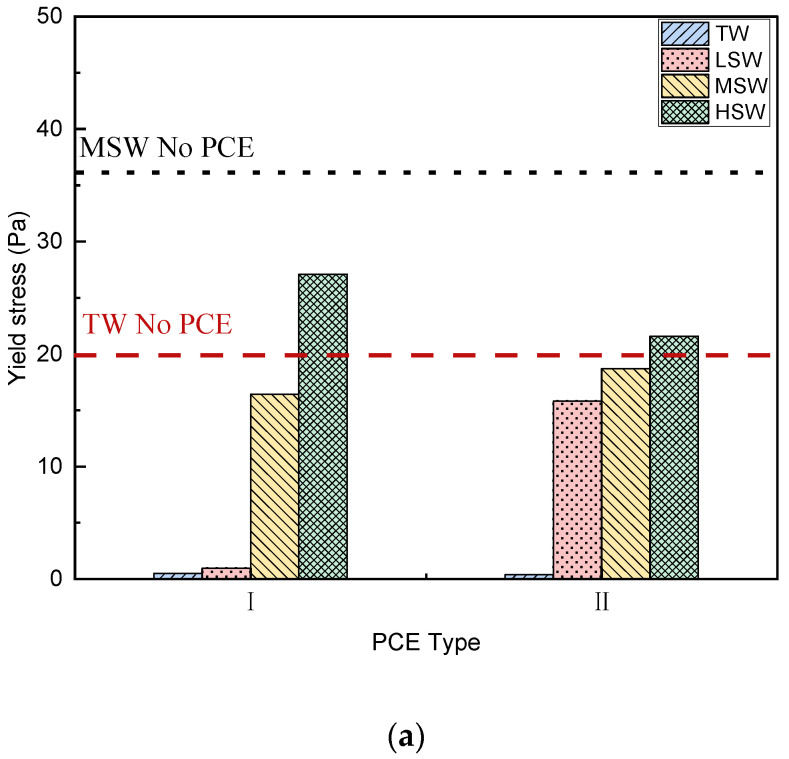

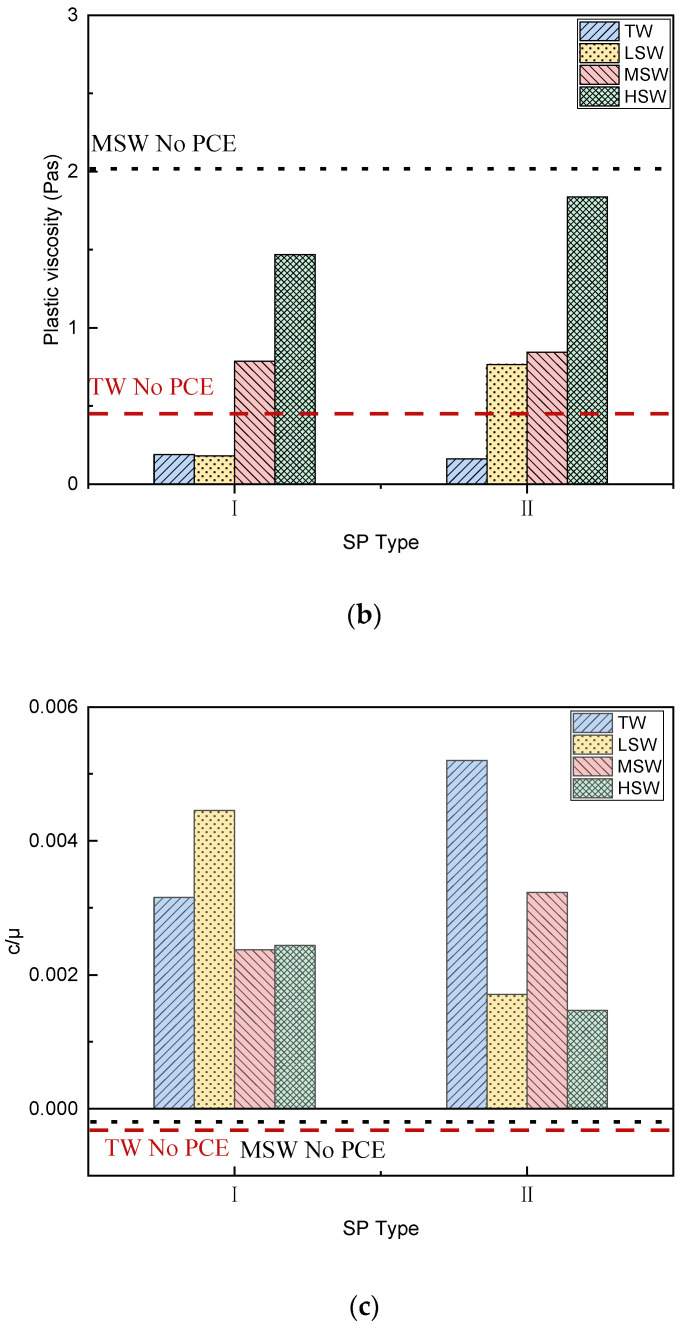
Effect of SW salinity fitted by the Modified Bingham model on rheological behavior of cement paste with the two PCEs (Equation (2)). (**a**) Yield stress; (**b**) Plastic viscosity; (**c**) c/μ value.

**Figure 10:**
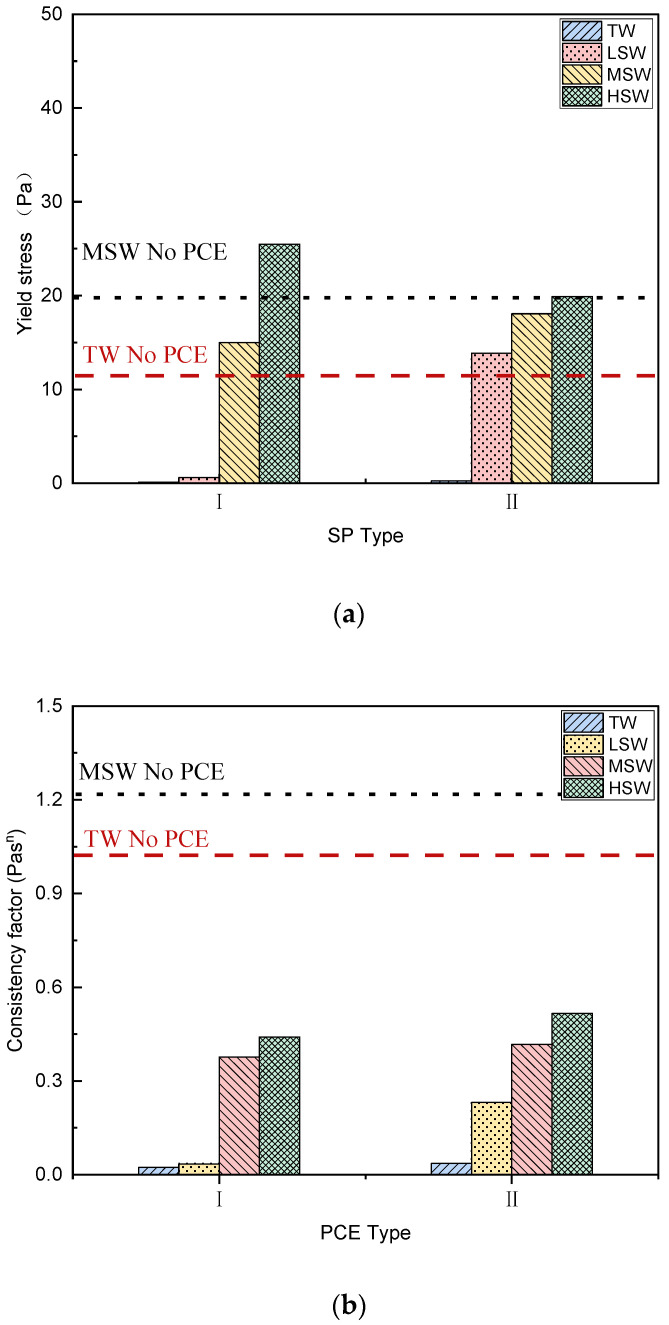

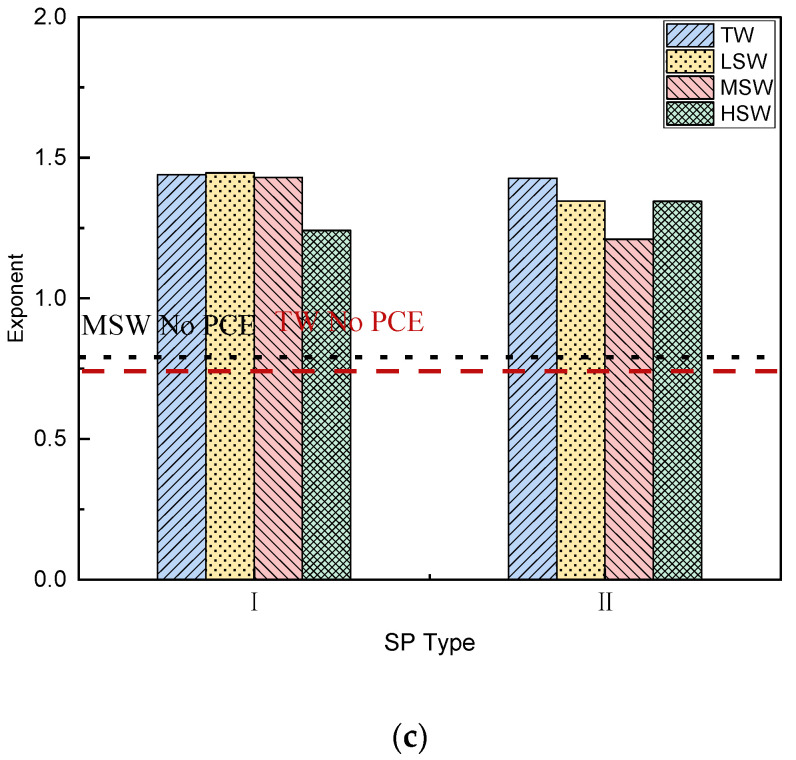
Effect of SW salinity fitted by the Herschel–Bulkley model on rheological behavior of cement pastes with the two PCEs (Equation (3)). (**a**) Yield stress; (**b**) Plastic viscosity; (**c**) Exponent.

In this section, different rheological models, namely the Bingham model (Equation (1) and Figure 8), the Modified Bingham (MB) model (Equation (2) and Figure 9), and the Herschel–Balkley model (HBM) (Equation (3) and Figure 10), are used to analyze the rheological properties of cement paste under the different salinity conditions and to verify the applicability of each model. 

##### Dynamic Yield Stress

The dynamic yield stresses based on the three rheological models are shown in Figure 8a, Figure 9a, and Figure 10a. As shown in Figure 8a, negative yield stress was observed in the cement paste, indicating a change in rheological behavior. Specifically, shear thickening occurred, as has been reported in PCE-mixed high-fluid cementitious materials [48,49]. In addition, when salinity increased from LSW to HSW, higher yield stress was observed. Similarly, when the MB model or HB model were applied, the influence from the PCE architecture and SW salinity on the yield stress was the same, except for the removal of the negative value, which could not happen in actual engineering. This is well corroborated by the minislump results presented in Figure 6 and will be discussed further below.

##### Plastic Viscosity

The plastic viscosity (consistency factor) based on the three rheological models is presented in Figure 8b, Figure 9b, and Figure 10b. As shown in all the figures, the plastic viscosity of cement paste decreased after adding PCEs, as calculated using each model. It is well-known that PCE decreases the yield stress and plastic viscosity of cement paste, which is consistent with many studies on PCE [50]. However, when SW was applied, the viscosity increased significantly with increasing salinity, and the viscosity was higher. This may be due to the rich ions in SW promoting hydration [26], as hydration products can bridge particles and form aggregates.

##### c/μ and Exponent

The rheological properties of the cement paste were changed after adding PCE [48]. The c/μ and exponent of the non-linear MB and HB models are plotted in Figure 9c and Figure 10c, respectively. As shown in Figure 9c, with the addition of the PCEs, the c/μ value increased from negative to positive, which suggests that shear thickening occurred with the addition of PCEs [51]. Shear thickening was also observed based on the HB model (Figure 10c), as the exponent increased to more than one [52]. Shear thickening normally happens under conditions of large solid and non-flocculated particles [49]. This means that when hydrodynamic forces overcome repulsive forces [53,54], temporary agglomerates can be formed. As a result, shear thickening can occur as the viscosity increases with increasing shear rate due to the enlargement of particle clusters [55]. 

#### 3.3.3. Relationship between Yield Stress and Minislump

**Figure 11:**
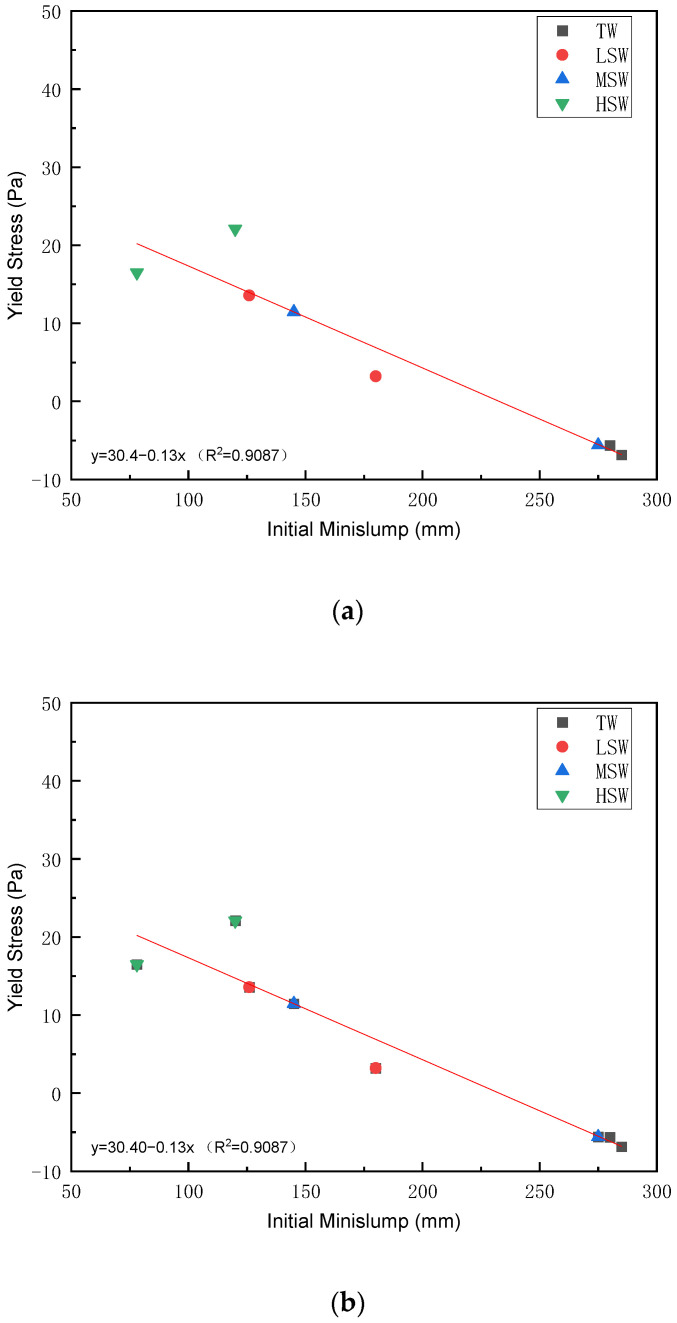

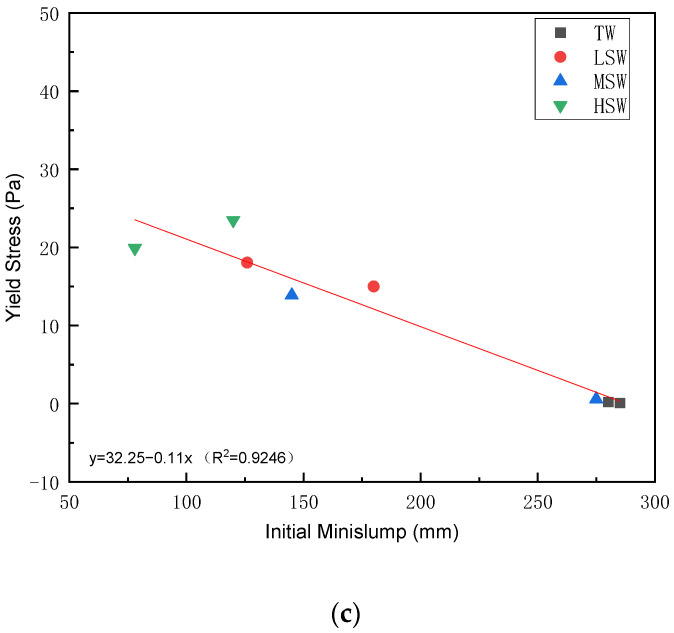
Relationship between yield and initial minimum slump stress fitted by the three models. (**a**) Bingham model; (**b**) Modified Bingham model; (**c**) HerschelBulkley model.

The relationship between the yield stress and minislump has been established by several previous studies [38,56]. Since plastic viscosity shows limited correlation with workability, the relationship between the dynamic yield stress and the minislump fitted by the three rheological models is explored in Figure 11.

As shown in Figure 11, a linear relationship with high R^2^ values between the initial minislump and yield stress was observed under all three applied models. This suggests a good prediction of workability by all three rheological models. However, a negative yield stress was observed in the Bingham model, indicating that the Bingham model was not suitable for the evaluation of the rheological behavior of cement with the PCEs. The high R^2^ values and the similarity between model results indicate that the MB and HB models can correctly describe this behavior.

### 3.4. Setting Time

**Figure 12:**
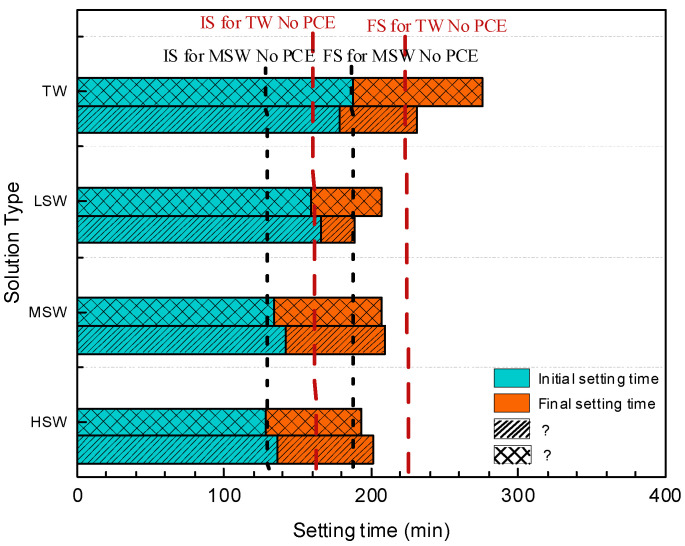
Effect of SW salinity on setting time of cement paste with the two PCEs.

The setting time of cement paste with TW and SW added with PCEs is presented in Figure 12. Clearly, the addition of PCE delayed the initial and final setting time of cement paste, and similar trends were observed in the study by Zhang et al. [57]. The reason for this could be that the complexation between the carboxyl group on the main chain of PCE and Ca^2+^ ions delayed the hydration of cement [44]. Moreover, it should be noted from Figure 11b that with increasing SW salinity, the setting time (especially initial setting) was accelerated due to the promoted hydration, which was similar to previous research [58]. For example, the initial setting time in LSW, MSW, and HSW was 166 min, 142 min, and 136 min, respectively, representing reductions rate of 7.3%, 20.6%, and 24.0% from the setting times in TW. This is because the rich ions (mainly Cl^−^) in seawater play a role in promoting hydration, which could be more intense at a higher salinity due to the increase in ion concentration.

### 3.5. Compressive Strength

**Figure 13:**
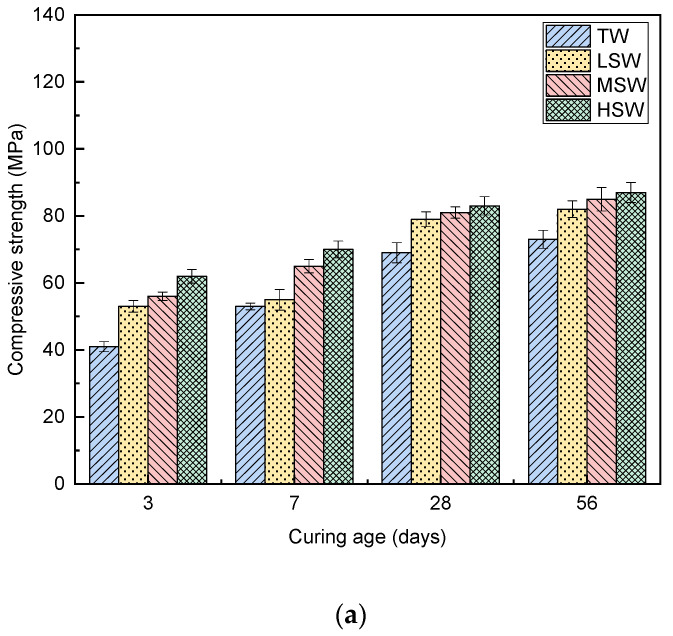

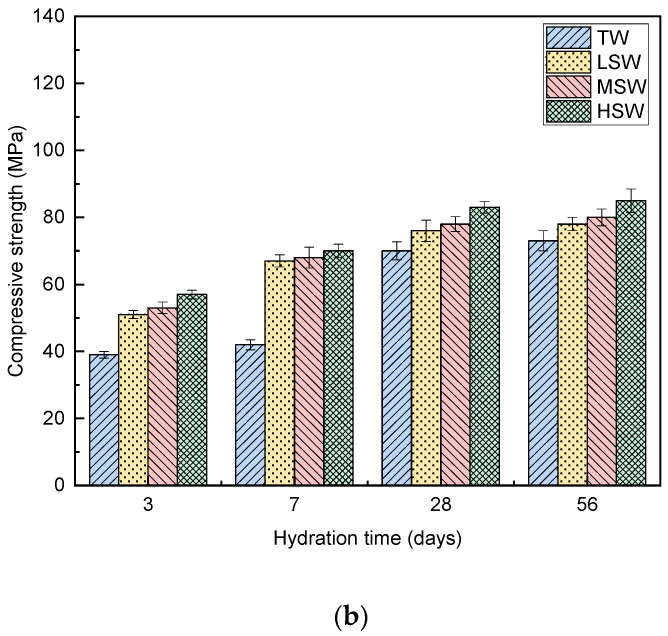
Effect of SW salinity on the compressive strength of cement paste with the two PCEs: (**a**) PCE-I; (**b**) PCE-II.

The effect of SW salinity on the compressive strength of cement paste with the two PCEs is presented in Figure 13. As shown in the figure, compared to TW, the introduction of SW significantly increased the compressive strength of the cement paste, which is consistent with the results obtained in previous studies [9]. Moreover, it should be noted that compressive strength increased with increasing salinity of seawater due to the acceleration of cement hydration by ions in seawater (Section 3.4).

### 3.6. Situation of PCE in Cement

#### 3.6.1. Adsorption Amount

**Figure 14:**
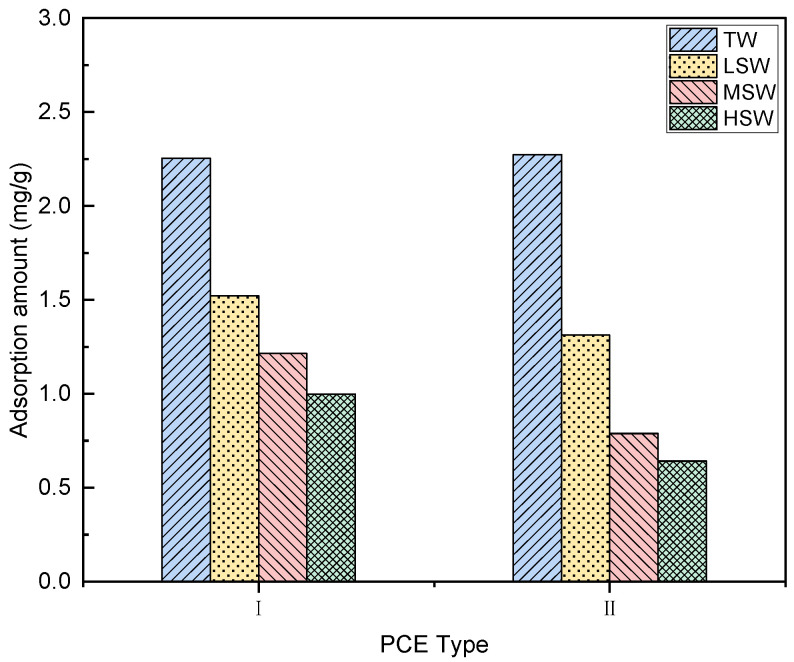
Effect of SW salinity on adsorption amount of cement paste with the two PCEs.

The adsorption amount of the PCEs in different solutions is presented in Figure 14. The increase in salinity, which resulted in the ions in the solution increasing, led to a reduction in the adsorption (Figure 13b). For example, for PCE-I, the adsorption amounts in TW, LSW, MSW, and HSW were 2.2543 mg/g, 1.5213 mg/g, 1.2137 mg/g, and 0.9987 mg/g, respectively, while those for PCE-II were 2.0718 mg/g, 1.3125 mg/g, 0.9894 mg/g, and 0.6412 mg/g, respectively. Hence, the reductions due to the introduction of LSW, MSW, and HSW were 32.51%, 46.16%, and 55.99%, respectively, for PCE-I, while higher reductions of 41.77%, 56.11%, and 71.56% were observed for PCE-II.

As presented in Table 2, regardless of the salinity, there are rich ions in SW, such as Na^+^, Cl^−^, and SO_4_^2−^. [59]. Obviously, the negatively charged ions (i.e., Cl^−^ and SO4^2−^) can lead to competitive adsorption and hence a reduced adsorption of PCE, which further reduces the dispersion ability of PCE [28,60]. It is widely accepted that Na^+^ and Mg^2+^ cations can promote the adsorption of PCE [61]. However, the formation of a Mg^2+^ complex may crosslink PCE molecules [47], which then reduces the performance of PCEs. Moreover, high acid–ether ratios generally indicate a higher charge density. Therefore, PCEs with higher acid–ether ratios should offer higher adsorption capabilities. However, lower adsorption was observed in the PCE with a short side chain, which could be due to the formation of a complex with divalent ions and a carboxylate group [31,32].

#### 3.6.2. Thickness of Adsorption Layer

**Figure 15:**
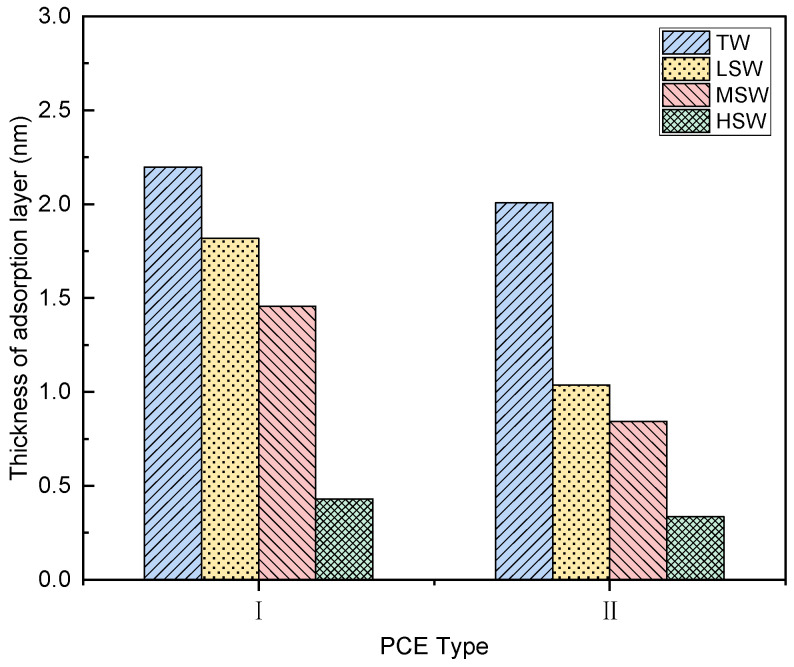
Effect of SW salinity on the thickness adsorption layer of cement paste with the PCEs.

The thickness of the adsorbed PCE layer was calculated according to the change of the Si2p XPS signal (Figure 15). The longer the side chain length, the thicker the PCE-I adsorption layer, which also confirms the reason proposed above for the good workability (Figure 6) and rheology (Figure 8a, Figure 9a, and Figure 10a) of PCE-I. This has been previously reported in a PCA-superplastic gel system [40]. The thickness of the adsorbed PCE layer is positively correlated with the dispersion capacity. Consistent with the expected behavior of adsorption capacity, when SW replaced TW, the thickness of the adsorption layer significantly reduced. As further illustrated in Figure 15b, increased SW salinity further decreased the thickness of the adsorbed PCE layer, and the reduction was more pronounced when high salinity was applied (HSW). The reasons for this could be that (1) the higher ion strengths in SW shrunk the conformation of the PCEs, which reduced the steric repulsions of the PCEs as demonstrated by Figure 5; (2) the accelerated hydration in SW-blended cement paste generated more hydration products, consuming the PCEs by intercalation actions [62].

#### 3.6.3. Water Film Thickness

**Figure 16:**
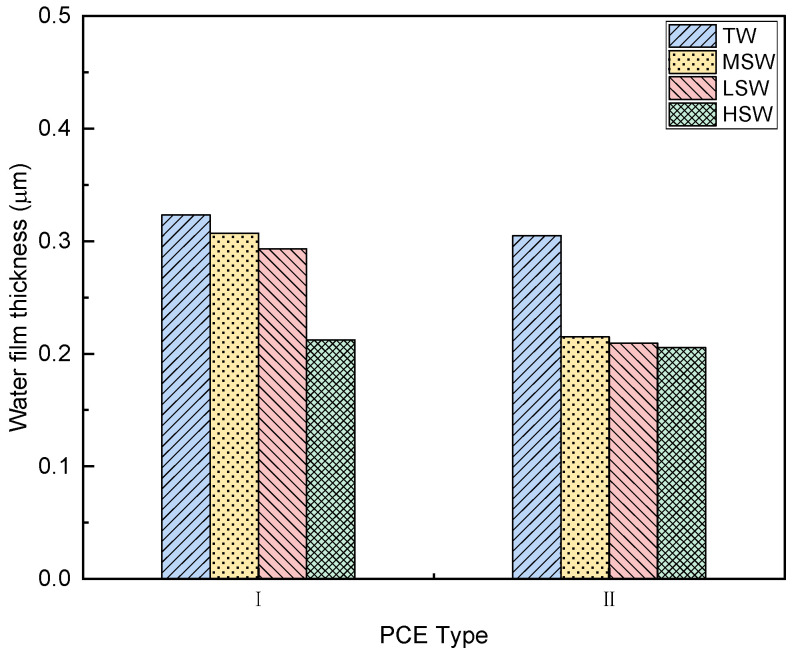
Effect of SW salinity on the water film thickness of cement paste with the two PCEs.

The water film thickness (WFT) of the cement paste is presented in Figure 16. Compared with TW, the WFT of all samples in SW was lower and showed a reducing trend with increasing ion concentration. This suggests that the lubrication of the cement particles was poor, so the workability was low. This may be because the increase in ions with early strength accelerated the hydration of the cement, and the water around was consumed faster.

## 4. Discussion

According to the experimental results presented here, the performance of PCE in SW-mixed cement is related to the type of PCE and the SW salinity. As described in Section 3.2 and Section 3.3, with increasing SW salinity, the dispersion and fluidity of PCE in cement paste decrease, while the thixotropic area, yield stress, and plastic viscosity increase. In general, PCE-I (with a high acid–ether ratio and a long side chain) and PCE-II (with a low acid–ether ratio and a short side chain) provided good performance in SW-mixed cement paste at any salinity.

Compared with TW, the large number of ions in SW (as shown in Table 2) did not lead to the degradation of the PCEs (Figure 3). However, it did change the dispersion of the PCEs. The hydrodynamic radius Rh decreased with the increasing salinity, with the smallest value being observed in HSW (Figure 5). As the dispersion performance of PCE was mainly determined by the steric hindrance generated by the side chain, the dispersion ability decreased with increasing salinity.

The adsorption amount of PCE and the thickness of the adsorption layer also affect the workability of cement paste. As described in the literature [38] and in Section 3.3.3, minislump is closely related to rheological parameters. The relationships seen here between the initial minimum slump of the cement slurry and the adsorption amount, the initial minimum slump and the adsorption layer thickness, and the initial minimum slump and the water film thickness were established to evaluate and explore the potential mechanism controlling the rheological parameters. It can be observed that the initial minimum slump of the cement slurry had a close linear relationship with the adsorption amount, the thickness of the adsorption layer, and the water film thickness. Hence, this can be applied to illustrate the performance of different PCEs in the cement-based system.

It is well-known that massive ions in SW, especially Mg^2+^ and SO_4_^2−^, significantly affect the adsorption behavior of PCEs [63]. This also suggested that SO_4_^2−^ can reduce the adsorption of PCE, while Mg^2+^ can promote the adsorption of PCE [38]. However, the formation of a Mg complex between the PCE molecule and its carboxylic acid group may be unfavourable to the adsorption of PCE [47]. In addition, it should be noted that in the presence of SO_4_^2−^, a rapidly forming Aft is positively charged and adsorbs PCE by electrostatic interaction. Meanwhile, improving the water resistance of cement particles can release trapped water, and the excess water can thicken the water film, lubricate the cement particles, and ultimately improve the workability of the paste. Since the main mechanism of PCE operation is based on spatial repulsion, the creation of a thicker barrier may facilitate the dispersion of cement particles. Therefore, PCE-I with the longer side chain and higher acid–ether content performed better than PCE-II.

## 5. Conclusions

This study systematically investigated the effects of polymer molecular structure and salinity of seawater on the performance of PCEs in seawater-blended cement paste. According to the results, the following conclusions can be drawn:

(1) The replacement of TW with SW did not lead to the obvious degradation or change of solubility of PCEs. However, the Rh values of the PCEs were significantly reduced, leading to weak steric hinderance.
(2) The PCEs with different molecular architecture exhibited different performance in cement paste blended with TW and SW. PCE-I with longer side chain and higher acid–ether content performed better than PCE-II.
(3) The rheological properties of the cement paste were changed after adding both PCEs. By comparing different rheological models, a non-linear model (i.e., a modified Bingham model and a Herschel–Bulkley model) provided a better description of the rheology of seawater-blended cement paste.
(4) SW reduced the dispersion performance of the PCE, but the highest adsorption amount and thickest adsorption layer were observed in cement paste with PCE-II with long side chain and high acid–ether ratio. With increasing SW salinity, the adsorption amount reduced, and the adsorption layer became thinner.
(5) The addition of PCEs and SW decreased the WFT due to the release of the excessive water. The decreased WFT resulted in the reduction in the workability of the cement paste.
(6) The increase in salinity accelerated the setting and increased the compressive strength of the specimen cement strength.

## Figures and Tables

**Table 1:** Summary of the chemical and mineral compositions of the cement used in this study (mass, %).

SiO_2_	Al_2_O_3_	CaO		Fe_2_O_3_	MgO	R_2_O	f-CaO	SO_3_		Loss	Cl^−^
21.12	5.23	3.58		64.44	2.11	0.52	0.95	0.90		1.40	0.03
C_3_S			C_2_S			C_3_A			C_4_AF		
58.93			14.42			7.81			10.88		

**Table 2:** Concentrations of principal ions in the TW and SW used in this study.

	Na^+^	K^+^	Ca^2+^	Mg^2+^	Cl^−^	SO_4_^2−^
TW	4.37	14.04	19.64	1.76	17.29	13.63
LSW	4.28 × 10^3^	256.64	152.62	616.43	0.87 × 10^4^	1.47 × 10^3^
MSW	1.05 × 10^4^	393.27	383.02	1.28 × 10^3^	1.66 × 10^4^	2.15 × 10^3^
HSW	1.37 × 10^4^	839.27	452.90	1.88 × 10^3^	2.78 × 10^4^	4.20 × 10^3^

Note: LSW—lower-salinity seawater. MSW—medium-salinity seawater. HSW—higher-salinity seawater.

**Table 3:** The synthesis parameters (molar ratio of the monomer and solid content) of the two PCEs synthesized and used in this study.

Code	AA	HPEG		Solid Content (%)
		2400	3000	
PCE-I	8.00		1.00	50.21
PCE-II	4.00	1.00		50.18

**Table 4:** Molecular weight of the two PCEs used in this study.

Type	Mn (Da)	Mw (Da)	PDI
PCE-I	30,125	54,918	1.82
PCE-II	24,416	42,900	1.76

Note: Mn—number of average molecular weight, Mw—weight average molecular weight, PDI—Polymer dispersity index, which can be calculated by Mw/Mn.

**Table 5:** Minislump of TW-mixed and SW-mixed cement paste with the two PCE types.

PCE Type	Solution	Minislump		
		5 min	30 min	60 min
PCE-Ⅰ	TW	285	297	270
	LSW	275	260	282
	MSW	180	100	58
	HSW	120	60	59
PCE-Ⅱ	TW	280	270	265
	LSW	145	105	85
	MSW	126	71	60
	HSW	78	65	59

## Data Availability

All the data associated with this study are available from the corresponding author upon request.
